# Scavenger Receptor Structure and Function in Health and Disease

**DOI:** 10.3390/cells4020178

**Published:** 2015-05-22

**Authors:** Izma Abdul Zani, Sam L. Stephen, Nadeem A. Mughal, David Russell, Shervanthi Homer-Vanniasinkam, Stephen B. Wheatcroft, Sreenivasan Ponnambalam

**Affiliations:** 1 Endothelial Cell Biology Unit, School of Molecular and Cellular Biology, University of Leeds, Leeds LS2 9JT, UK; 2 Leeds Vascular Institute, Leeds General Infirmary, Great George Street, Leeds LS1 3EX, UK; 3 Leeds Institute of Cardiovascular and Metabolic Medicine, University of Leeds, Leeds LS2 9JT, UK

**Keywords:** Scavenger receptor, Oxidized LDL particle, Atherosclerosis, Signal transduction

## Abstract

Scavenger receptors (SRs) are a ‘superfamily’ of membrane-bound receptors that were initially thought to bind and internalize modified low-density lipoprotein (LDL), though it is currently known to bind to a variety of ligands including endogenous proteins and pathogens. New family of SRs and their properties have been identified in recent years, and have now been classified into 10 eukaryote families, defined as Classes A-J. These receptors are classified according to their sequences, although in each class they are further classified based in the variations of the sequence. Their ability to bind a range of ligands is reflected on the biological functions such as clearance of modified lipoproteins and pathogens. SR members regulate pathophysiological states including atherosclerosis, pathogen infections, immune surveillance, and cancer. Here, we review our current understanding of SR structure and function implicated in health and disease.

## 1. Introduction

**Figure 1:**
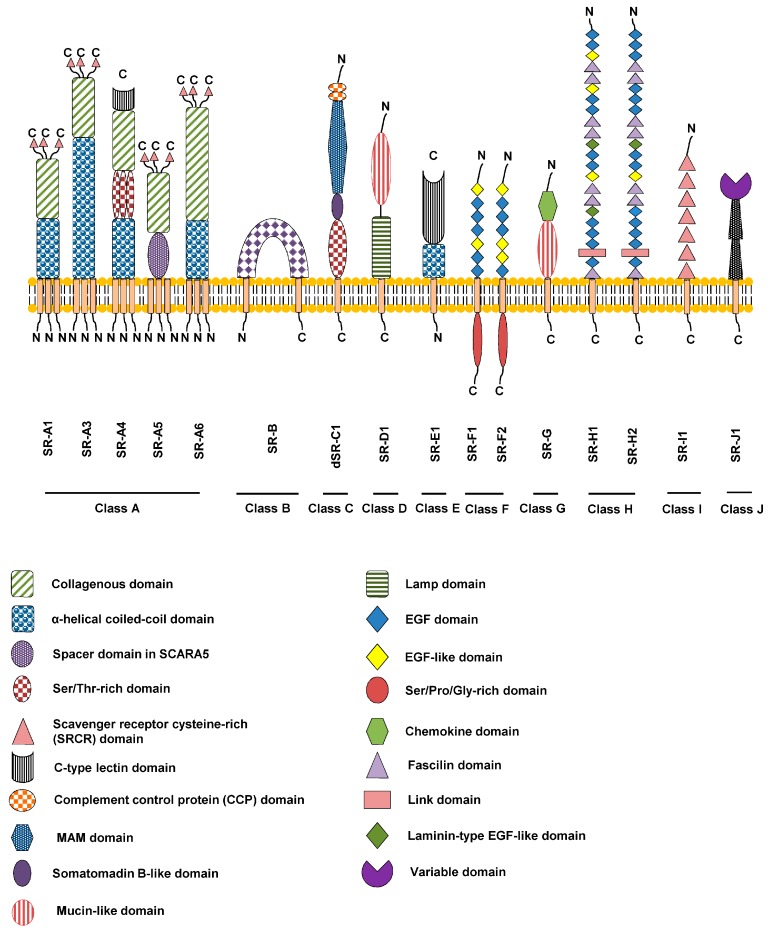
Schematic overview of the SR membrane protein supergroup. The different classes are denoted A-J and specific domains are denoted by the codes shown. All SR classes have mammalian orthologues except Class C (dSR-C1) which can only be found in insects.

Ground-breaking studies that led to the identification of scavenger receptor (SR) in macrophages were first described by Brown and Goldstein in the 1970s. It was found that modified oxidized low-density lipoprotein gets internalized and degraded, but not native LDL. SRs comprise a diverse array of integral membrane proteins and soluble secreted extracellular domain isoforms. We have termed these proteins as belonging to the ‘SR supergroup’ (Figure 1), as opposed to a superfamily, as this latter term implies primary sequence similarity shared across the whole supergroup. A key point is that SR members within each class bear primary sequence similarity but different classes bear little or no primary sequence similarity. The common feature uniting this disparate group of proteins within the SR supergroup is their ability to recognize common ligands such as polyionic ligands including lipoproteins, apoptotic cells, cholesterol ester, phospholipids, proteoglycans, ferritin, and carbohydrates. SRs were initially identified on basis of their biochemical ability to recognize and bind different modified forms of LDL e.g. oxidized LDL (OxLDL), and such interactions can promote macrophage differentiation into foam cells leading to chronic conditions such as atherosclerosis. The term and classification for scavenger receptors used in this context is standardized according to the recent review by Prabhudas *et al.* [1]. Based on our current understanding of SR structure and biological function, we have grouped these proteins into Classes A-J (Figure 1) [2].

## 2. Class A

### 2.1. Genetics, Protein Structure and Expression

These are Type II membrane proteins of ~400-500 residues with an N-terminus comprising a short cytoplasmic domain followed by a single transmembrane region and a large extracellular domain that mediates ligand recognition (Figure 1). A unique feature of Class A proteins is a collagen-like domain with collagen-binding activity with homotrimers of SR-A at the cell surface [3]. Members include SR-A1, SR-A3, SR-A4, SR-A5 and SR-A6.

The *SR-A1* (*SCARA1*) gene is on chromosome 8 in both mice and humans. SR-A1 is relatively abundant on macrophages but also present on vascular smooth muscle and endothelial tissues, especially when endothelial cells experience oxidative stress [4] or upon exposure to phorbol esters [5]. One common feature is exemplified by SR-A1 such as the ability to bind modified or oxidized LDL particles. The *SR-A3* (*SCARA3)* gene is present on human chromosome 8; gene transcription is stimulated by oxidative stress [6]. The *SR-A5* (*SCARA5)* gene on human chromosome 8 is also present in other mammals, birds and fishes. SR-A5 is expressed in epithelial, testis, heart and brain tissues and is a receptor for ferritin-bound iron; however, it does not appear to bind modified LDL particles but plays a functional role in innate immunity [7].

The *SR-A4* (*SRCL)* gene is located on human chromosome 18 and gene expression is stimulated by oxidative and hypoxic stress. SR-A4 contains a C-type lectin domain and is widely expressed including placenta, umbilical cord, lung, skeletal muscle and heart. The *SR-A6 (MARCO)* gene is on human chromosome 1 [8]; the gene product lacks the α-helical coiled-coil domain present in other Class A members [9]. SR-A6 is expressed in tissues of the peritoneum, lymph nodes, liver and spleen macrophages. Bacteria or bacterial lipopolysaccharide (LPS) can both stimulate SR-A6 expression [10], linking its function to the innate immune response to bacterial infection [11]. However, SR-A6 lacks the ability to bind modified LDL particles.

### 2.2. Signal Transduction, Trafficking and Cell Function

**Figure 2:**
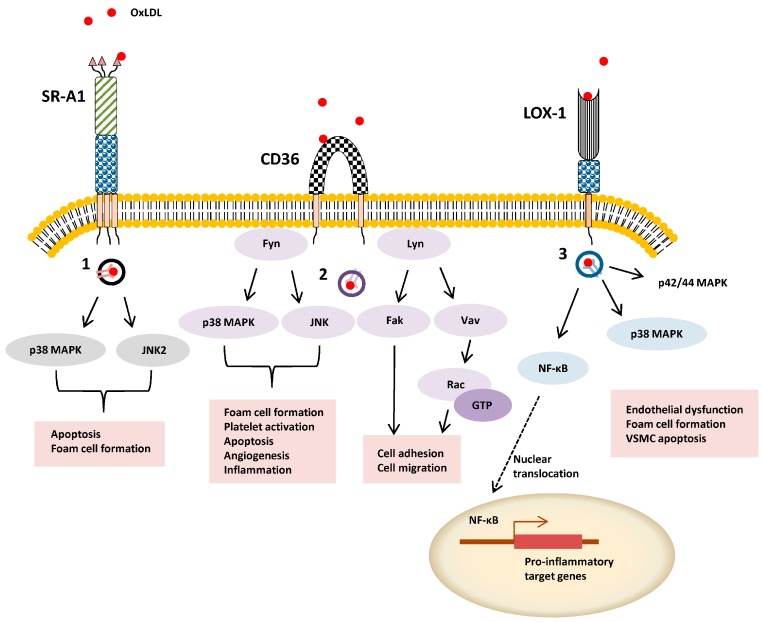
Schematic overview of ligand-stimulated SR signal transduction. OxLDL-stimulated activation of intracellular signaling pathways is exemplified by SR-A, SR-B2 (CD36) and SR-E1 (LOX-1). Different endocytosis pathway are denoted **1**–**3** (**1**) caveolae-mediated uptake, (**2**) lipid-raft dependent uptake, and (**3**) clathrin-independent pathway.

SR-A1 can undergo internalization from the plasma membrane via clathrin-dependent endocytosis (CDE) or clathrin-independent endocytosis (CIE) routes. SR-A1 binding to modified LDL is linked to CDE via recognition of a cytoplasmic dileucine motif [12]. One such example of CIE is caveolae-mediated uptake: SR-A1-ligand internalization via this route stimulates apoptosis [13] (Figure 2). In antigen-presenting cells, SR-A1-mediated pathogen uptake involves phagocytosis by a lipid raft-dependent mechanism [14]. SR-A1-null mice display 50-70% reduction in acetylated LDL (AcLDL) and OxLDL uptake with a corresponding size reduction in atherosclerotic lesions [15,16]. Nonetheless, there is agreement that gene knockouts cause reduced pro-inflammatory responses, macrophage apoptosis and cellular necrosis with better stabilization of atherosclerotic plaques [17,18]. Interestingly, viral gene therapy promotes soluble SR-A1 expression and secretion decreased modified LDL accumulation, foam cell incidence and atherosclerosis [19].

In macrophages, the c-Jun N-terminal kinase 2 (JNK2) protein is activated in SR-A1-mediated foam cell formation [20]. Nonetheless, SR-A1-null macrophages display elevated pro-inflammatory responses including increased p42/44 mitogen-activated protein kinase (MAPK) phosphorylation, NF-κB nuclear translocation and increased secretion of TNFα, IL-6 and IFNβ [21]. Alveolar macrophage SR-A1 or SR-A6 can mediate clearance of more complex oxidized lipids in lung tissues [22]. One view is that SR-A1 and SR-A6 mediates rapid pro-inflammatory ligand internalization on vascular cells thus reducing interactions with TLRs [23]. However, SR-A1 and SR-A6 appear to signal through different intracellular pathways with distinct effects on immune responses such as IL-12 production [24]. On a phenotypic level, expression of SR-A6 in cultured cells stimulates development of plasma membrane-derived dendrites and lamellipodia [25], key membranous structures which mediate pathogen engulfment by eukaryote cells. Mice lacking both SR-A1 and SR-A6 expression display altered spleen morphology, significantly lowered circulating IgM and IgG3 antibody levels for bacterial surface polysaccharides [26], thus linking leukocyte function to a pathogen-specific immune response. SR-A1 can bind HSP70 members to promote antigen uptake and processing for presentation to the adaptive immune response [27,28].

SR-A4 also belongs to the collectin family of pattern recognition receptors which are implicated in innate immune responses. During the pro-inflammatory response at sites of infection, SR-A4 can mediate recognition of complex carbohydrates and neutrophil granule glycoproteins [29,30]. SR-A4 levels closely mirror the cellular ability to bind, internalize and process bacterial and yeast pathogens [31,32]. SR-A5 could act as a tumor suppressor by binding focal adhesion kinase (FAK); such interaction inhibits activation of the FAK-Src-Cas pathway that is linked to hepatocellular carcinoma development and progression [33]. Increased SR-A5 expression causes inactivation of STAT3, a key transcriptional regulator in pro-inflammatory gene expression [34]. SR-A5 expression can also confer cell recognition of bacterial pathogens [35]. SR-A5 has been implicated in functioning as a renal receptor for ferritin for endocytosis and delivery of this iron-containing ligand to specific kidney tissues [7].

## 3. Class B

### 3.1. Genetics, Protein Structure and Expression

The members of this class of gene products usually contain 450–500 residues and comprise of SR-B1, SR-B2 and SR-B3. These three members have 2 transmembrane regions located close to the N- and C-termini which straddle a central domain of ~400–450 residues that is glycosylated and mediates ligand recognition (Figure 1). The short N- and C-terminal cytosolic regions are implicated in regulatory roles in signal transduction and trafficking. The *SR-B1* (*SCARB1*) gene is on human chromosome 12. The SR-B1 gene product binds HDL, viruses and bacteria; mutations or allelic variations in *SR-B1* are associated with an increased risk of atherosclerosis, infertility and/or an impaired innate immune response [36,37,38]. The *SR-B3* (*SCARB2*) gene is on human chromosome 4 and expressed in liver, brain, heart and macrophages; the SR-B3 protein mediates binding to HDL particles [39,40]. The *SR-B2* (*CD36*) gene is on human chromosome 7 and widely expressed. SR-B2 has many functions including macrophage OxLDL uptake to promote foam cell formation, platelet activation/aggregation, apoptosis, angiogenesis, inflammation; its levels are elevated by a fat-rich diet, inflammation and oxidative stress [41,42,43].

### 3.2. Signal Transduction, Trafficking and Cell Function

CD36 (SR-B2) binds a variety of ligands including thrombospondin-1, oxidized phospholipids/lipoproteins, long-chain fatty acids, modified lipid particles, apoptotic cells, bacterial and fungal pathogens [44]. OxLDL binding to SR-B2 triggers intracellular signaling events which inhibit macrophage migration [45]. SR-B2 is enriched within cholesterol-rich membrane microdomains and interacts with other receptors such as tetraspanins and integrins. Activated SR-B2 signal transduction involves the tyrosine kinase Fyn, p38 MAPK and JNK (Figure 2). SR-B2 activation is also implicated in FAK activation and altered cell adhesion and migration. Notably, SR-B2-mediated intracellular signaling through Fyn is implicated in phosphorylation and activation of Vav proteins. These act as guanine nucleotide exchange factors for Rho and Rac GTPases that are implicated in actin remodeling, membrane dynamics and cell migration. SR-B2 can bind microbial diacylglycerides to stimulate a pro-inflammatory TNFα response upon bacterial infection. Furthermore, SR-B2 acts as a co-receptor to TLR4-TLR6, which augments the pro-inflammatory signaling in response to OxLDL [46]. SR-B2 is crucially implicated in regulating the host malarial response; monocytes, macrophages and dendritic cells expressing SR-B2 can help to clear the malarial pathogen from the circulation [47]. However, vascular endothelial SR-B2 binding to infected red blood cells enables the parasite to escape immune clearance.

SR-B1 can bind and internalize AcLDL or OxLDL particles [48]. SR-B1-mediated HDL uptake can be dynamin-independent [49]. SR-B1-mediated endocytosis causes relatively mild OxLDL degradation [50], suggesting key differences to other SR-mediated ligand delivery to lysosomes. Human SR-B1 overexpression in rabbits increased HDL clearance but also caused higher plasma LDL levels [51]. In the liver, SR-B1 may mediate HDL uptake and endocytosis; however, in peripheral tissues, SR-B1 binding to HDL may stimulate cholesterol efflux from internal stores [52]. SR-B3 can act as a viral pathogen receptor including Enterovirus 71 and Coxsackie virus [53,54] via CDE and recognition of a cytoplasmic dileucine motif [55]. Mice lacking SR-B3 show a reduced immune response to bacterial infection with lowered production of pro-inflammatory chemokines and cytokines [56].

## 4. Class C

Class C SRs are expressed only in insects such as fruit flies and mosquitoes and are involved in the innate immune response against pathogens such as bacteria. This occurs by a mechanism called pattern recognition whereby pathogen-derived ligands that contain a characteristic repetitive molecular pattern bind to cell surface receptors thus triggering a sustained immune response. Class C proteins (320–629 residues) are either Type I membrane proteins or soluble secreted proteins where the extracellular domains contain N-proximal compliment control protein (CCP) region preceding a MAM motif (Figure 1). SR-C1 and SR-C2 are membrane-bound but SR-C3 and SR-C4 are soluble secreted proteins [57]. SR-C2, SR-C3 and SR-C4 are expressed during insect embryonal, larval and pupal stages. In fruit flies, lack of the Class C protein dSR-C1 reduced phagocytosis of fungal and bacterial pathogens [57], showing a widespread conservation of SR innate immunity function during evolution. The Class C receptor such as SR-C1 is a pattern recognition receptor for bacteria; however, SR-C1 expression in mammalian cells can confer binding, internalization and degradation of AcLDL [58].

## 5. Class D

The *SR-D1 (CD68)* gene is on human chromosome 17 and expressed on cells associated with the immune system and bone marrow such as monocytes, macrophages, dendritic cells and osteoclasts. Phorbol esters, OxLDL or GM-CSF elevate whereas bacterial LPS or TNFα inhibit SR-D1 levels [59,60], showing a link between inflammation and SR-D1 function. The human SR-D1 gene product is a Type I membrane protein of 354 residues which is heavily glycosylated, exhibiting greatly reduced SDS-PAGE mobility. SR-D1 contains an N-proximal mucin-like domain, a proline-rich hinge region followed by a lysosome-associated membrane protein (LAMP) homology domain, a single transmembrane region and a short 12-residue cytoplasmic domain [61] (Figure 1). SR-D1 can bind OxLDL, lectins, selectins and also mediate phagocytosis and bone resorption [62,63]. When SR-D1 is expressed on monocytes, it promotes OxLDL binding and uptake [62], suggesting a role in leukocyte-mediated effects in atherosclerosis. Furthermore, soluble recombinant SR-D1 delivery into a mouse model reduced foam cell incidence and abdominal aortic plaque development with increased plaque stabilization [64]. However, SR-D1 depletion showed little change in OxLDL-mediated atherosclerosis in mice [65] and OxLDL binding and accumulation is unchanged in SR-D1-null mouse macrophages [66]. The role of SR-D1 in the immune response is currently not clear. Deletion of the mouse SR-D1 gene did not impair macrophage ability to deal with bacterial challenge (innate immunity); this also did not impair cytokine production and actually enhanced antigen presentation (adaptive immunity) [66].

## 6. Class E

### 6.1. Genetics, Protein Structure and Expression

The SR-E1 also known as lectin-like oxidized low-density lipoprotein receptor (LOX-1/OLR1/SR-E1), which is on human chromosome 12 within a region enriched for genes involved in the innate immune response. Human SR-E1 is a Type 2 membrane protein of 273 residues which comprises a short N-terminal cytoplasmic domain, a single transmembrane region followed by an extracellular domain containing a coiled-coil ‘neck’ region and a C-type lectin-like domain (Figure 1). Although initially identified as an endothelial-specific receptor for OxLDL, low SR-E1 levels are present on the endothelium, vascular smooth muscle and immune cells. SR-E1 binds a variety of ligands including OxLDL, phosphatidylserine, and bacteria; SR-E1 binding to the heat-shock protein HSP70 enables dendritic cells to present antigens on MHC Class I for immune surveillance [67,68].

*SR-E1* is a non-essential gene and SR-E1-deficient mice are healthy; however, the incidence of atherosclerosis is drastically reduced in an LDL-R and *SR-E1* double-knockout transgenic mouse model [69]. Polymorphisms within the human *SR-E1* locus is linked to increased CVD risk [70], but many of these are non-coding mutations that do not affect SR-E1 protein sequence nor function. Most cells in adult tissues display low or negligible levels of SR-E1 due to regulation by shear stress and pro-inflammatory signals (e.g. TNFα, superoxide, glucose). Stable overexpression of SR-E1 in transgenic mice stimulated inflammatory intramyocardial vasculopathy [71]. However, transient viral gene therapy stimulated liver-mediated OxLDL clearance and reduction in atherosclerosis in a mouse model [72]. Such findings have led to much debate as to the role of SR-E1 as a protective or pro-atherogenic factor in inflammation and atherosclerosis.

### 6.2. Signal Transduction, Trafficking and Cell Function

SR-E1 recognition and binding of OxLDL stimulates pro-inflammatory signaling promoting endothelial dysfunction, foam cell phenotype and vascular smooth muscle apoptosis [73,74]. OxLDL binding to SR-E1 can stimulate activation of different protein kinases including p42/44 MAPK and p38 MAPK [75] (Figure 2). In addition, SR-E1-mediated signal transduction causes NF-κB activation [76], a key feature of pro-inflammatory response in immune and vascular cells. Overexpression of the SR-E1 transgene in cultured cells stimulates bacterial binding [77]. SR-E1-mediated intracellular signaling during bacterial infection stimulates inflammation, cytokine production and increased neutrophil recruitment which in turn promotes immune suppression, with a net consequence of increased mortality [68]. Both SR-A1 and SR-E1 levels are elevated in response to bacterial infection or challenge [78]. A key feature of SR-E1 activation is stimulation of SR-E1 gene expression; pro-inflammatory stimuli such as glucose and TNFα also have similar effects. SR-E1 and SR-F1 can both bind *Enterobacteriaceae* membrane proteins which triggers activation of the key Toll-like receptor family member (TLR2) that regulates many aspects of the innate immune response [79].

## 7. Class F

The Class F group contains SR-F1 (SREC1) and SR-F2 (SREC2). *SR-F1* is on human chromosome 17 whereas SR-F2is on human chromosome 22. Human SR-F1 and SR-F2 are Type 1 membrane proteins of 850-900 residues, an extracellular domain of ~450 residues containing multiple EGF-like repeats, a single transmembrane region and a relatively large cytoplasmic domain of ~400 residues (Figure 1). SR-F1 is present on neuronal and endothelial cells in heart, lung, ovary and placenta; pro-inflammatory cytokines such as TNFα inhibit SR-F1 expression [80]. SR-F1 binds to carbamylated LDL (cLDL), AcLDL or OxLDL particles. However, SR-F2 lacks SR activity but preferentially forms heterodimers with SR-F1 [81]. Such SR-F1/SR-F2 heterodimers lose the capacity to mediate lipid particle recognition suggesting that SR-F2 suppresses the ligand-binding properties of SR-F1. The Class F members can both regulate modified LDL binding and uptake but their effects on atherosclerosis initiation and progression are unclear. SR-F1 not only recognizes a wide variety of modified lipid particles but also heat-shock protein HSP90 complexes; receptor-ligand complexes can undergo CDE and delivery to the endosome-lysosome system [82,83].

## 8.Class G

The Class G protein SR-G (SR-PSOX) is also called chemokine 16 (CXCL16). Human SR-G is a Type 1 membrane protein of 254 residues with an N-terminal extracellular domain, a single transmembrane region and a short cytoplasmic domain (Figure 1). The SR-G extracellular domain mediates endocytosis of phosphatidylserine or OxLDL, and delivery to endosome-lysosome system. The SR-G extracellular domain contains a chemokine-related motif followed by a mucin-like stalk region. Cleavage within this mucin-like region by disintegrin-like metalloproteases (ADAMs) causes ‘shedding’ of a soluble secreted SR-G. Human *SR-G* is on chromosome 17 and expressed on vascular smooth muscle cells, endothelial cells, monocytes, macrophages, kidney podocytes and in atherosclerotic lesions [84]. SR-G has important innate immunity functionality through recognition of bacteria and CpG-rich DNA found in other pathogens [85,86]. SR-Gis only expressed in smooth muscle cells and macrophages within atherosclerotic plaques and on the endothelium within cardiac valves during inflammatory valvular disease [87,88,89]. Manipulation of SR-G levels modulates macrophage differentiation into foam cells [90,91], suggesting a pro-atherogenic function. Polymorphisms within the SR-G gene are linked to coronary artery stenosis [92]. SR-Glevels can be upregulated to recruit CD4+ T cells to affected sites during inflammatory disorders in various tissues [87,93,94]. Mice lacking SR-G produced lowered cytokines and liver natural killer cells [95]. This molecule may play a vital role not only in recruiting but also promoting interaction of both T and natural killer cells to dendritic cells [96].

## 9. Class H

The SR-H1 and SR-H2 membrane proteins are Fasciclin, EGF-like, laminin-type EGF-like and link (FEEL) domain-containing scavenger receptors, which are Type 1 membrane glycoproteins or soluble secreted glycoproteins of up to 2570 residues (Figure 1). *SR-H1* and *SR-H2* are on human chromosome 3 and 12, respectively. The SR-H gene products are expressed by cells from the spleen, lymph nodes, macrophages, bone marrow, foetal liver and adult liver endothelial cells. The SR-Hextracellular or soluble domains have three blocks containing two Fasciclin domains interspersed with EGF-like domains and laminin EGF-like domains and a single Fasciclin domain adjacent to the transmembrane domain. SR-H1 and SR-H2 bind to AcLDL, advanced glycation end-products (AGE) and bacteria. SR-H1 expression on monocyte has been postulated to be a biomarker for increased CVD risk [97].

SR-H1 promotes bacterial recognition bacteria and stimulates lymphocyte diapedesis through lymphatic and vascular endothelial cell monolayers. SR-H1 also stimulates recruitment of CD4+ FoxP3-positive regulatory T-cells, indicating an important role in the immune response to pathogen infection [98,99]. The expression of SR-H1 in the fetus suggests a role in immune system development during embryogenesis [100].

SR-H2 has a classical NPxY-like endocytic motif within its cytoplasmic domain whereas SR-H1 contains cytoplasmic acid-rich motifs that could function as non-classical endocytic motifs. SR-H1 levels can be elevated by sorting nexin 17 (SNX17) [101], suggesting such an interaction may promote efficient endosome-plasma membrane recycling thus preventing SR-H1 degradation within lysosomes. Macrophage SR-H1 mediates recognition of matricellular secreted protein acidic and rich in cysteine (SPARC), a potent angiogenesis inhibitor, to promote endocytosis and lysosomal degradation [102]. SR-H1 can also promote the clearance of apoptotic and necrotic bodies [103,104]. Macrophages can express SR-H2 to promote phagocytosis and clearance of aged cells, apoptotic bodies and heparin-linked proteins, showing functional similarities to other SR members.

## 10. Class I

### 10.1. Genetics, Protein Structure and Expression

Class I scavenger receptor comprise of SR-I1 (CD163) that is primarily restricted to the hematopoietic cell lineage. This receptor is classified as the Class I scavenger receptor because of the presence of type B scavenger receptor cysteine-rich (SRCR) domain, which are encoded by a single exon and containing eight cysteine residues. SR-I1 is a 130 kDa transmembrane Type 1 membrane glycoprotein that is primarily expressed in monocytes and macrophages. *SR-I1/CD163 (M130)* was mapped to human chromosome 12p13. The primary structure of SR-I1 displays an extracellular domain composed of nine SRCR domains in tandem, a transmembrane region followed by a short intracellular cytoplasmic tail. It has been reported that the SR-I1primary mRNA undergoes alternative mRNA splicing to produce 5–6 splice variants and gene products [105,106]. There are at least 3 different splice variants that exhibit differing cytoplasmic C-termini: the shortest 7 residue cytoplasmic domain is the most abundant and is largely present at the plasma membrane, whilst the longer C-terminal variants of 42 and 147 residues are preferentially localized to the *trans*-Golgi network and endosomal compartments [106]. SR-I1is also known as “hemoglobin scavenger receptor” due to its important role in mediating Hb recognition and clearance in tissue macrophages [107,108].

SR-I1 is readily cleaved in the plasma forming soluble molecules due to the presence of exofacial proteolytic sites [109]. Interestingly, the cleaved fragments of these molecules function differently from their membrane-bound precursors. For example, the proteolytic fragment of SR-I1has the extracellular domain intact and has been shown to associate with iron; thus it has the ability to prevent growth of bacterial pathogens [109]. SR-I1 is also present as soluble forms although their functional role is unknown; it has been suggested that these soluble fragments could be potential biomarkers for inflammatory and autoimmune diseases [110,111,112].

### 10.2. Signal Transduction, Trafficking and Cell Function

As suggested from its functional name as a hemoglobin scavenger receptor, SR-I1 helps the removal of haptoglobin-hemoglobin (Hp-Hb) complexes via the heme oxygenase-1 (HO-1) pathway to reduce pro-inflammatory haem in the circulation [108]. This indicates the role of SR-I1 in anti-inflammatory response by mediating the uptake of toxic haem in macrophages [107]. Apart from its function as a scavenger receptor, SR-I1 has been shown in animal studies to be expressed on bone marrow macrophages to initiate growth and survival of erythroblast. SR-I1also has a role as a PAMP receptor. It has been reported that SR-I1 binds to both Gram-negative and positive bacteria [113]. SR-I1 has also been reported to be involved in intracellular signaling such as phosphorylation of protein kinase C (PKC) [106].

## 11. Class J

### 11.1. Genetics, Protein Structure and Expression

The sole member of SR-J1 is the receptor for advanced glycation end-products (RAGE). SR-J1 is a 32 kDa multi-ligand transmembrane receptor that belongs to the immunoglobulin gene superfamily. SR-J1 is reported to express in endothelial cells, hepatocytes, smooth muscle cells and monocytes [114]. Its expression can be down-regulated in pathological conditions. Full-length SR-J1is composed of an extracellular V (variable)- type domain, a single transmembrane spinning helix that connects the short C-terminal cytosolic domain and two C-type domains. SR-J1 is a pattern recognition receptor that this receptor is able to interact and be activated by a number of pro-inflammatory ligands such as β-amyloid [115], S100/calgranulin [116], phosphatidylserine [117] and high-mobility group protein 1 (HMGB1) [118]. Under physiological conditions, the expression of SR-J1 is low, but can be aggravated in response to chronic conditions. These pro-inflammatory endogenous molecules are involved in inflammation and physiological stress.

### 11.2. Signal Transduction, Trafficking and Cell Function

The up-regulation of SR-J1 expression by these pro-inflammatory ligands has a positive effect in a scenario where inflammation occurs; unlike most receptors their expression is down-regulated in chronic inflammatory condition. Moreover, most of these ligands are secreted by hematopoietic cells, therefore these cells depend on the expression and propagation of SR-J1 in respond to inflammation [119,120]. AGE-bound SR-J1 is implicated in signal transduction mediating processes such as oxidative stress, apoptosis and inflammation [121]. Stimulation of SR-J1 is also involved in neuronal differentiation and cell migration especially during development. Upon ligand stimulation of SR-J1, the pro-inflammatory gene expression is activated by NF-κB translocation into the nucleus; *SR-J1* is itself a target of NF-κB thus providing a positive feedback loop to amplify the response. Indeed, it has been reported that NF-κB is also activated by AGEs [119].

Furthermore, *in vitro* studies showed the activation of MAPK signaling cascades through SR-J1-mediated activation by AGE. This triggered the oxidative stress pathway, which in turn led to activation of NF-κB [122]. Another pathway involved in the inflammatory signal transduction is the MAPK pathway JNK. From an *in vitro* study, expression of the pro-inflammatory marker protein vascular cell adhesion molecule-1 (VCAM-1) was decreased by JNK inhibition [123]. Additionally, activation of JNK by SR-J1ligands also showed to increase the transcriptional activity of activator protein-1 (AP-1), which increases expression of pro-inflammatory genes.

## 12. Biological Roles of SR

### 12.1. SR and ROS Production

The association of SR in a number of cardiovascular-related diseases is highly anticipated, nonetheless the mechanism for such receptors have not yet been fully understood. A variety of studies indicate a functional link between scavenger receptors and reactive oxygen species (ROS), a key pro-inflammatory signal and early event in atherosclerosis. ROS molecules rapidly diffuse and modify proteins, phospholipids, carbohydrates and nucleic acids to mediate significant changes in cell physiology. Most evidence linking ROS production to scavenger receptor function is from studies on the SR-E1 (LOX-1) and SR-B2 (CD36). Binding of OxLDL to SR-E1 stimulates ROS production in the endothelium and vascular smooth muscle [124]. SR-E1-activated ROS production is implicated in causing oxidative DNA damage providing an explanation for increased cell apoptosis. One aspect of SR-E1-stimulated ROS production is the reduction in intracellular nitric oxide levels [125] thus stimulating vasoconstriction and hypertension upon increased oxidative stress. During ischemia-reperfusion injury, mice lacking SR-E1 exhibited decreased oxidative stress [126], suggesting that SR-E1 expression stimulates cardiac dysfunction. Evidence suggests that OxLDL activation of endothelial SR-E1, elevated ROS levels and intracellular signaling through NF-KB are functionally linked [127].

Although Class A SRs are not functionally linked to ROS production, increased oxidative conditions is implicated in blocking SR-A1-mediated ligand uptake [128]. The Class B scavenger receptor SR-B2 is activated by oxidized phospholipids: SR-B2-deficient mice display significantly less ROS levels in vascular walls suggesting that SR-B2 regulates ROS levels in vascular smooth muscle cells [129]. SR-B2 expression is elevated in ischemic brain tissues with SR-B2-deficient mice showing reduced ROS levels and neural damage [130].

### 12.2. SR and Apoptosis

SR function is increasingly linked to apoptosis in a wide variety of cell types. SR-A1, SR-B1 and SR-B2 (CD36) binding to apoptotic bodies promotes phagocytosis and clearance [131]. SR-A1 overexpression in aortic VSMCs can promote apoptosis [132]. Binding of fucoidan ligand by the macrophage SR-A1 triggers endocytosis by clathrin-dependent and caveolae-dependent pathways; however, caveolae-dependent uptake and signaling stimulates apoptosis via a p38 MAPK and JNK-dependent intracellular signaling pathway [13]. Expression of SR-A6 (MARCO) in macrophages can mediate silica particle binding and endocytosis with increased apoptosis [133]. SR-A3 (SCARA3) induces apoptosis by binding and inactivating cleavage and polyadenylation-specific factor 3, a key RNA-processing enzyme [134]. In contrast, SR-A3expression provides protection from apoptosis by removing ROS. HDL-stimulated SR-B1 activity is also anti-apoptotic [135]. In vascular cells, thrombospondin-1 activation of SR-B2triggers downstream signaling through p38 MAPK and caspase-dependent pathways with increased apoptosis [136].

SR-E1 is implicated in regulating apoptosis in vascular and non-vascular cells. SR-E1 levels are linked to coronary artery endothelial cell apoptosis, with OxLDL further stimulating SR-E1 expression [137]. This pathway potentially involves p38 MAPK, NF-κB, Bax/Bcl-2 and caspase activity [74,138]. SR-E1 function is linked to apoptosis in the endothelium, VSMCs, macrophages, epithelial cells and neurons. In addition, SR-E1 can mediate recognition of activated platelets and apoptotic bodies, through a phosphatidylserine-dependent mechanism [139,140]. Both SR-H1 (FEEL-1) and SR-H2 (FEEL-2) are also implicated in regulating apoptotic cell body phagocytosis and clearance in thymocytes and macrophages [141,142]. This may involve recognition of exposed phoshatidylserine on the outer membrane of apoptotic bodies. There is little known about whether other scavenger receptor classes can promote apoptosis in different cells and tissues.

### 12.3. SR and Angiogenesis

Increasingly, many scavenger receptors are linked to cancer development and progression. One view is that SR proteins regulate pro-inflammatory responses and such phenomena are dysregulated in cancer. For example, SR-A1 may negatively regulate anti-tumor immunity [143]. SR-A1 may promote tumor progression in ovarian and pancreatic cancer [144]. SR-A3 is implicated to function as a tumor suppressor in prostate cancer; however, SR-A3 expression is increased in ovarian carcinoma [145,146]. Elevated SR-I1 (CD163) levels are also linked to increased breast and rectal cancer and poor patient prognosis [147,148]. Raised levels of SR-BI may increase prostate cancer progression [149]. There is a strong link between SR-B2 and cancer by providing pro-apoptotic and anti-proliferative signals. However, the function of SR-B2 in tumor angiogenesis and growth is likely to be context-dependent [150].

SR-E1 activation may also promote angiogenesis and tumor progression [151]. SR-E1 expression in transformed epithelial cells stimulates intracellular signaling and cell migration and tumor development [152,153]. SR-H1 (FEEL-1) is also implicated in tumor metastasis: migratory cancer cells can bind to the lymph vessel endothelium in a SR-H1-dependent manner [154]. SR-F1 (SREC-1) is implicated in binding HSP110 and GRP170 to stimulate tumor-derived antigen uptake and processing for a better immune response to tumors [155].

## 13. Concluding Remarks

Discoveries on SR function in multicellular organism homeostasis, development and function has been increasing on a daily basis. Even though SRs had been initially identified on the basis of their ability to bind modified LDL particles, now it is clear that this property alone can no longer limit the classification of SRs. These proteins not only been implicated in atherosclerosis, but also in inflammation, the immune response and other chronic diseases such as cancer and diabetes. Comprehensive studies on intracellular trafficking and signal transduction on SR family are rather limited; however, there are evidence reviewed here the response upon ligand binding implicate on receptor internalization and trafficking. Atherosclerosis causes a wide range of pathologies ranging from heart attacks, strokes to peripheral arterial disease. A central feature of this condition is the migration of monocytes into arterial vessel walls, development into macrophages and differentiation into foam cells that die and form the core of the atherosclerotic lesion. Rupturing of these plaques or lesions causes blood clot or thrombus formation, arterial occlusion and the clinical symptoms. More work is needed to unravel their potential medical field. Functional SR manipulation using small molecule inhibitors, antibodies or gene therapy may help to target chronic, acquired and inherited diseases.

## Abbreviations

SR: scavenger receptor
OxLDL: oxidized low-density lipoprotein
HDL: high-density lipoprotein
CDE: clathrin-dependent endocytosis
CIE: clathrin-independent endocytosis
AcLDL: acetylated low-density lipoprotein
MAPK: mitogen-activated protein kinase
JNK: c-Jun N-terminal kinase
FAK: focal adhesion kinase
AGE: advanced glycation end-products
PAMP: pathogen-associated molecular pattern
ROS: reactive oxygen species
VSMC: vascular smooth muscle cell
TLR: Toll-like receptor
